# Deep Learning-Based Mpox Skin Lesion Detection and Real-Time Monitoring in a Smart Healthcare System †

**DOI:** 10.3390/diagnostics15192505

**Published:** 2025-10-01

**Authors:** Huda Alghoraibi, Nuha Alqurashi, Sarah Alotaibi, Renad Alkhudaydi, Bdoor Aldajani, Joud Batawil, Lubna Alqurashi, Azza Althagafi, Maha A. Thafar

**Affiliations:** Department of Computer Science, College of Computers and Information Technology, Taif University, Taif 21944, Saudi Arabia; hudaghoribie@gmail.com (H.A.); nuhasalqurashi@gmail.com (N.A.); sarahsinhatalotaibi@gmail.com (S.A.); renadalkhudaydi@gmail.com (R.A.); bdoorabdulrazagaldajani@gmail.com (B.A.); joodbatawil@gmail.com (J.B.); lubnaalqurashi@gmail.com (L.A.); azza.althagafi@tu.edu.sa (A.A.)

**Keywords:** AI, Mpox detection, deep learning, CNN, vision transformer, smart healthcare

## Abstract

**Background/Objectives:** Mpox, a viral disease marked by distinctive skin lesions, has emerged as a global health concern, underscoring the need for scalable, accessible, and accurate diagnostic tools to strengthen public health responses. This study introduces ITMA’INN, an AI-driven healthcare system designed to detect Mpox from skin lesion images using advanced deep learning. **Methods:** The system integrates three key components: an AI model pipeline, a cross-platform mobile application, and a real-time public health dashboard. We leveraged transfer learning on publicly available datasets to evaluate pretrained deep learning models. **Results**: For binary classification (Mpox vs. non-Mpox), Vision Transformer, MobileViT, Transformer-in-Transformer, and VGG16 achieved peak performance, each with 97.8% accuracy and F1-score. For multiclass classification (Mpox, chickenpox, measles, hand-foot-mouth disease, cowpox, and healthy skin), ResNetViT and ViT Hybrid models attained 92% accuracy (F1-scores: 92.24% and 92.19%, respectively). The lightweight MobileViT was deployed in a mobile app that enables users to analyze skin lesions, track symptoms, and locate nearby healthcare centers via GPS. Complementing this, the dashboard equips health authorities with real-time case monitoring, symptom trend analysis, and intervention guidance. **Conclusions**: By bridging AI diagnostics with mobile technology and real-time analytics, ITMA’INN advances responsive healthcare infrastructure in smart cities, contributing to the future of proactive public health management.

## 1. Introduction

On 11 March 2020, the World Health Organization (WHO) declared the novel coronavirus (COVID-19) outbreak a global pandemic. Calling it “the first pandemic of the 21st century” because it spread quickly from continent to continent, causing more than 8000 infections in 8 months—with a 10% case fatality ratio [1]. Recently, a new public health risk emerged when a multi-country Mpox outbreak was reported to the WHO by several non-endemic countries. On 14 September 2022, about 103 member states from six regions reported 59,147 confirmed cases of Mpox and 22 deaths. Given the current outbreak of Mpox cases, the WHO has declared it a global health emergency [2]. Dealing with epidemic diseases such as Mpox and limiting their spread is critical to maintaining the public health of the entire population. Relying only on traditional clinical examinations can be time-consuming, not easily accessible, and cause an overload on the medical professionals and hospitals. Therefore, there is a pressing need for an intelligent, effective, and scalable diagnostic system for Mpox disease to support early detection, reduce transmission, and assist both individuals and healthcare providers [3].

Recent advances in artificial intelligence (AI)—particularly in machine learning (ML), deep learning (DL), and computer vision—have enabled promising solutions across various domains, especially in medicine and healthcare [4,5,6,7,8,9]. These technologies have played a significant role in automating medical image analysis tasks, such as object detection, image segmentation, and image classification [10,11]. In particular, convolutional neural networks (CNNs) and transformer-based models have demonstrated impressive performance in classifying various skin lesions, including skin cancer detection and dermatological disease recognition [12,13]. Beyond classification, DL has also advanced image segmentation and enhancement tasks critical for clinical applications. For instance, Curti et al. [14] proposed a semi-supervised active learning framework for wound image segmentation, reducing annotation requirements while ensuring accurate clinical assessments, highlighting the feasibility of learning paradigms beyond fully supervised training. Similarly, Griffa et al. [15] reviewed mobile computer-vision applications for automatic ulcer segmentation, underscoring the translational potential of deploying AI-driven segmentation tools directly on smartphones for real-world dermatology and wound care. Moreover, Fiscone et al. [16] demonstrated that an enhanced deep super-resolution model, originally designed for natural images, generalized effectively to brain MRI, evidencing the adaptability and transferability of DL models across imaging modalities and tasks beyond classification. Complementing these efforts, recent work by Alsinan et al. [17] further illustrates how CNN- and transformer-based approaches continue to enhance diagnostic accuracy in dermatological imaging, reinforcing the broader role of AI in advancing automated disease recognition. However, despite the growing urgency for effective diagnostic tools, the application of these models to Mpox detection remains limited in scope, performance, and real-world deployment.

This research addresses these challenges and proposes an end-to-end smart healthcare system for Mpox detection and tracking by integrating DL and other AI and computer vision techniques. The system consists of three key components: First, a DL-based classification model trained on skin lesion images to accurately detect Mpox disease. Second, a smart mobile application that deploys an AI-based model for real-time diagnosis and symptom tracking with extra features. Third, a monitoring dashboard to visually represent Mpox cases and their geographical spread detected by the mobile application, allowing health authorities to respond rapidly and prevent disease outbreaks. This system also aims to reduce hospital overload by providing a preliminary, fast, and accurate diagnosis at home through the image-based diagnosis system, thus reducing hospital visits and saving resources for more critical cases. It also limits disease transmission and treats patients as quickly as possible by directing them to the nearest health center if the infection is confirmed, which preserves citizens’ public health and safety.

This study is particularly relevant in the context of Saudi Arabia’s Vision 2030, which emphasizes the integration of AI and digital health technologies to enhance the efficiency of healthcare services. By aligning with these national objectives, the proposed system aims to support the development of smart health infrastructure within the broader framework of smart cities. Our goal is to offer an accessible, low-cost, and AI-driven solution that enables early diagnosis and supports disease surveillance.

The key contributions of this research are summarized as follows:

- We developed ITMA’INN, an AI-powered healthcare system for early detection and monitoring of Mpox disease from skin lesion images with efficient, accurate, and fast diagnostic capabilities, supporting the healthcare system and preventing disease transmission.
- The system supports both binary classification (Mpox vs. non-Mpox) and multiclass classification (Mpox, Chickenpox, Measles, Cowpox, hand-foot-mouth disease (HFMD) and Normal), leveraging fine-tuned pretrained deep learning models including ViT, TNT, Swin Transformer, MobileViT, ViT Hybrid, ResNetViT, VGG16, ResNet-50, and EfficientNet-B0.
- We developed and implemented a user-friendly and cross-platform mobile application that enables users to upload skin lesion images for real-time AI-based diagnosis, track symptoms, receive guidance on the nearest healthcare centers, and access relevant and up-to-date disease information.
- We created a real-time monitoring dashboard to support healthcare authorities by visualizing case distribution, tracking patients’ trends and patterns, and facilitating data-driven decision-making in response to potential outbreaks.

The remainder of this research paper is structured as follows. Section 2 reviews the existing literature on Mpox detection using deep learning models. Section 3 describes the datasets utilized and provides a detailed explanation of the proposed methodology. Section 4 outlines the development of the mobile application and the dashboard. Section 5 presents the experimental setup and evaluation metrics. Section 6 discusses the results and key findings. Section 7 concludes the study and highlights potential future directions.

## 2. Related Works

Recently, researchers have paid more attention to developing image-based AI systems to identify Mpox and other viral skin-related infections [18,19,20], especially with the rapid advancement of AI that assists the development of such diagnostic systems. ML, DL, and transfer learning techniques have emerged as promising tools to capture the characteristics of skin lesions, facilitating Mpox disease detection [3,21,22,23].

With the emergence of transfer learning, several studies have applied CNN-based pretrained models for binary and multiclass classification of skin lesion images. One of these studies is the Mpox detection using a deep neural network (DNN) [3]. The authors utilized a dataset consisting of Mpox, Chickenpox, Measles, and Normal skin images. They explored two scenarios: a binary classification task (Mpox vs. non-Mpox) and a multiclass classification task (Mpox, Chickenpox, Measles, and Normal skin) to differentiate between skin diseases that exhibit visually similar lesions. They evaluated seven CNN-based pretrained models, which are InceptionResNetV2, InceptionV3, ResNet-152V2, VGG16, VGG19, Xception, and DenseNet-201. Those models prove their efficiency in image classification tasks. The result showed that the DenseNet-201 model outperformed the other models using several evaluation metrics, achieving an accuracy of 97.63% in the two-class scenario and 95.18% in the four-class scenario. They also employed interpretability techniques like Local Interpretable Model-agnostic Explanations (LIME) and Gradient-weighted Class Activation Mapping (Grad-CAM), which helped visualize the image regions that influenced the model’s decision. Another recent study published in 2023 [24] employed DL to diagnose Mpox from skin lesion images. Using a publicly available dataset, this study evaluated five pretrained CNN models: GoogLeNet, Places365-GoogLeNet, SqueezeNet, AlexNet, and ResNet-18. After performing hyperparameter optimization, ResNet-18 obtained the best accuracy of 99.49%. The modified version of the model also performed well, with accuracies above 95%. ResNet-18 was particularly effective due to its residual connections, which helped mitigate the vanishing gradient problem and allowed the model to focus on critical image features. LIME was also utilized here to explain the reasoning behind the classifier’s predictions. Interestingly, the Vision Transformer (ViT-B/16) showed extremely lower performance than CNN-based models on the same dataset. This poor performance highlights the challenges of employing transformer-based models in medical image classification, particularly when the dataset is limited in size or diversity. Nonetheless, in contrast to those findings, our study demonstrates that with suitable preprocessing, data augmentation, and fine-tuning techniques, transformer-based models (i.e., ViT, MobileViT, and TNT) can outperform standard CNNs in both binary and multiclass Mpox classification tasks.

Several other studies have extended the investigation of pretrained models through transfer learning, focusing on hyperparameter tuning and real-world deployment, building on earlier CNN-based methods and advanced alternative architectures. For instance, Murat Altun and coauthors [25] utilized other advanced and more complex CNN-based pretrained models and applied them in a transfer learning fashion for Mpox detection. These models are MobileNetV3-s, EfficientNetV2, ResNet-50, VGG19, and DenseNet-121. They utilized AUC, accuracy, recall, loss, and F1-score metrics to evaluate and compare the different models. The study investigated several hyperparameter values for optimization, such as the batch size, number of epochs, image size, layer count, activation function, optimizer, and loss function. The optimized hybrid MobileNetV3-s model achieved the best score, with an average F1-score of 0.98, AUC of 0.99, accuracy of 0.96, and recall of 0.97. Despite this study’s effectiveness and high performance, it still faced some limitations, including potential overfitting due to limited dataset diversity and the need for further real-world testing to ensure generalizability across different populations and skin types.

Similar to the previous study, Jaradat and coauthors [26] expanded this approach by incorporating additional CNN-based pretrained models in image-based Mpox identification. They investigated five popular CNN pretrained models: MobileNetV2, VGG19, VGG16, ResNet-50, and EfficientNetB3, and then fine-tuned them in a transfer learning approach. The results showed that the MobileNetV2 model performed the best, achieving an accuracy of 98.16%, a recall of 0.96, a precision of 0.99, and an F1-score of 0.98. The study highlighted the practicality of these models for rapid and accurate clinical diagnosis, especially when integrated into mobile platforms as a real-time assessment tool to detect Mpox cases. Likewise, another study [27] explored the development of DL algorithms to detect Mpox from skin images named MPXV-CNN. The MPXV-CNN model was trained using dermoscopic and clinical images, accurately distinguishing between different skin diseases. The MPXV-CNN method employed several CNN architectures, such as ResNet-18, ResNet-34, ResNet-50, ResNet-152, DenseNet-169, and VGG19_bn, and applied them in a transfer learning style. In the experiment, the authors utilized data augmentation to increase the size and diversity of the training data. The study adopted cross-validation for evaluation. The model was trained on 130,000 images and achieved 90% accuracy. They were able to integrate the MPXV-CNN method into a smartphone application. They developed a free, open-source application called PoxApp that allows users to take photos of lesions, answer questions, and get a risk assessment within five minutes. The app aims to enhance accessibility for communities with limited healthcare resources, encouraging individuals to pursue medical attention. Notably, the app can detect Mpox at different stages of the disease and offers users five levels of advice, ranging from “no action required” to “immediate consultation with a doctor”. Users can also submit their results to contribute to research efforts, helping scientists predict potential waves in Mpox infections and establish an early warning system. The advantages of this technology include increased diagnostic precision, scalability, and rapid screening capabilities. However, it is essential to address potential drawbacks, such as biases stemming from limited or imbalanced datasets, as well as the necessity for further validation across diverse clinical settings.

The last study [28] presented an Android mobile application that utilized DL techniques to analyze skin lesion images. The application has been developed using Android Studio and Java programming language. The system captures video input, extracts frames, and classifies lesions using several CNN-based pretrained models. Among six evaluated CNN models, the best-performing model was MobileNetv2. Moreover, the application includes the feature of making a quick initial diagnosis and can also classify images with 91.11% accuracy. In addition, the average inference times were observed to range from 19 ms to 831 ms. Despite the success of this application and promising results, the authors discussed some weaknesses, such as the limited size of the Mpox skin lesion images in the dataset, which caused an imbalance of dataset issues and thus affected the performance.

As shown in Table 1, which summarizes the task, dataset, splitting methodology, evaluation metrics, and best-performing models of the studies mentioned above, several methods have been developed for Mpox disease detection using different DL techniques, but there is still significant room for improvement. Most existing studies have employed CNN-based models, with limited exploration of Vision Transformers and their advanced variants, which are known for their strong performance in image classification tasks. Furthermore, few works have integrated these models into practical, real-time systems that combine accurate detection with mobile accessibility and public health monitoring. This study addresses several gaps by developing an AI-powered solution that leverages state-of-the-art transformer-based pretrained models, a user-friendly mobile application, and a real-time monitoring dashboard to support both individual diagnosis and broader epidemic tracking.

## 3. Methodology

### 3.1. Datasets

In this study, we utilized three publicly available datasets to train and evaluate deep learning (DL) models for Mpox detection and classification. The datasets include: MSLD, used for binary classification (Mpox vs. non-Mpox), MSLDv2.0, used for multiclass classification (six classes), and MSID, used for multiclass classification (four classes) and as an external test set. 

All datasets were obtained from the Kaggle repository and curated for skin lesion classification related to Mpox infections.

#### 3.1.1. Binary Classification Dataset (MSLD)

The binary classification task utilizes the Mpox Skin Lesion Dataset (MLSD) [29]. This dataset consists of two classes: Mpox and non-Mpox. The dataset is a collection of 228 original images divided into 102 images for the Mpox and 126 images for the other classes (i.e., non-Mpox). The non-Mpox class comprises skin lesion images of chickenpox and measles, selected for their visual similarity to Mpox lesions. The dataset is publicly accessible through the Kaggle repository given in the data availability statement section. Augmentation was performed by the dataset authors using MATLAB R2020a, applying a wide range of geometric and photometric transformations, including rotation, translation, reflection, shear, hue adjustments, saturation changes, contrast variation, brightness jitter, noise injection, and scaling. This augmentation process enhanced dataset variability, mitigated class imbalance, and improved training robustness. Both the original and augmented datasets were organized into separate directories to maintain a clear distinction between raw and synthetic data. The statistics of this dataset are shown in Table 2.

#### 3.1.2. Multiclass Classification Dataset (MSLD v2.0)

For the multiclass classification task, we used the Mpox Skin Lesion Dataset Version 2.0 (MSLD v2.0) [30], which comprises 755 original skin images collected from 541 distinct patients, ensuring a diverse and representative sample. The dataset is categorized into six classes: Mpox (MKP), Chickenpox (CHP), Measles (MSL), Cowpox (CWP), Hand-Foot-Mouth Disease (HFMD), and Healthy skin. According to the dataset creators, it has been endorsed by professional dermatologists and approved by the appropriate regulatory authorities. The dataset is publicly available and accessible through Kaggle, as given in the data availability statement. The original and augmented images are provided in separate folders to ensure reproducibility. As with MSLD, the augmentation performed by the dataset authors using MATLAB R2020a included geometric and photometric transformations such as rotation, translation, reflection, shear, color adjustments, noise addition, sharpening, blurring, elastic deformation, brightness adjustment, and scaling. This process expanded the training set to 7,208 images, substantially increased the size and diversity of the datasets, and contributed to improved model accuracy. The statistics of the original and augmented data for each class are presented in Table 3.

#### 3.1.3. Multiclass Classification Dataset II: MSID

The Monkeypox Skin Images Dataset (MSID) was introduced in the MonkeyNet study for Mpox detection using a deep CNN [31] and was collected from various internet-based sources and curated by the Department of Computer Science and Engineering, Islamic University, Bangladesh. This dataset includes a total of 770 original skin lesion images, distributed across four diagnostic classes: Monkeypox, Chickenpox, Measles, and Normal (Healthy skin). The dataset is publicly available and can be accessed via the Kaggle repository (see the data availability statement).

In our study, this dataset was utilized in two different experimental setups: as a standalone multiclass dataset (four classes) to train and test models independently, and as an external validation set to evaluate the generalization ability of models trained on the MSLDv2.0 dataset, thereby testing real-world deployment potential. 

Unlike the development datasets, no data augmentation was applied to MSID. All images were kept in their original resolution and form to ensure unbiased evaluation. The class distribution of MSID is provided in Table 4.

### 3.2. ITMA’INN System Overview and General Framework

**Figure 1:**
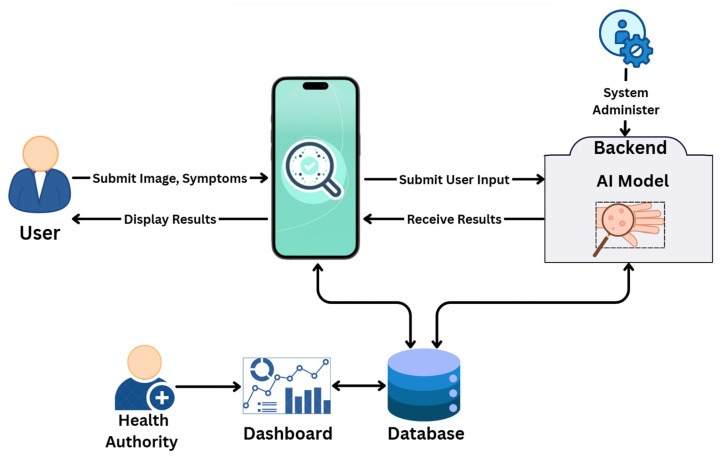
The Workflow of the ITMA’INN system, illustrating user input, AI-based lesion analysis, and real-time result delivery through the mobile application and dashboard.

Figure 1 presents the overall architecture of the proposed ITMA’INN system for Mpox diagnosis and tracking. The workflow can be summarized in the following six key components:

1. **Patient interactions with the mobile application:** Users can access the mobile application as registered users or continue as guests. The patient can upload an image of the suspected skin lesions for Mpox detection and optionally input symptoms for analysis.
2. **Data Transmission and Preprocessing:** Uploaded data is transmitted securely to the backend, where image processing techniques are applied to the images to enhance quality and ensure compatibility with the DL-based model.
3. **DL-Based Image Classification:** The preprocessed image is fed into a fine-tuned DL model based on pretrained vision transformers or CNN, which predicts the likelihood of Mpox infection (binary or multiclass).
4. **Diagnosis and User Feedback:** The model’s prediction, along with the confidence score, is displayed to the user on the application screen. The patients can also receive some recommendations and educational content.
5. **Health Authority Dashboard:** A real-time dashboard, synchronized with the app database, allows public health authorities to monitor Mpox case trends, track geographic spread, and support timely interventions.
6. **System Administration:** Backend operations, including performance monitoring, user management, and system maintenance, are handled by the system administrator.

We explained each of these steps and components in more detail in the corresponding sections.

### 3.3. AI-Model Pipeline

**Figure 2:**
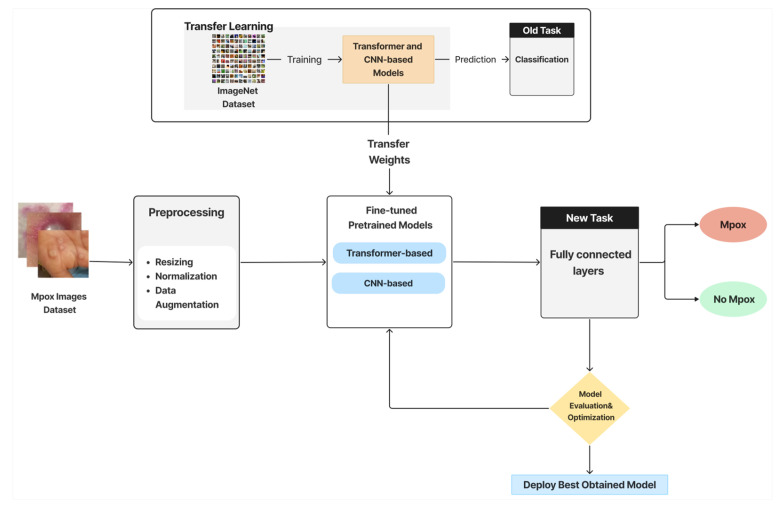
Schematic representation of the AI-based pipeline for Mpox classification using transfer learning with transformer-based and CNN-based pretrained models.

The AI model development pipeline for Mpox detection involves four primary stages, each critical to ensuring accurate and reliable classification of skin lesion images. Figure 2 illustrates the pipeline, showing each stage, which can be explained as follows:

The pipeline of building an AI model for Mpox detection involves four primary stages, each critical to ensuring accurate and reliable classification of skin lesion images. Figure 2 illustrates the pipeline, showing each stage, which can be explained as follows:

1. **Image Preprocessing and Augmentation:** This initial stage consists of 2 steps. First, preprocessing entails a few crucial steps to guarantee model compatibility, including image enhancement, resizing (to match model input dimensions), normalization, and optional cropping. Data augmentation is the second step, which is employed to boost the training data’s volume and diversity.
2. **Feature Extraction via Pretrained Models and Transfer Learning:** Pretrained DL models are employed to extract relevant features from images automatically. These models, originally trained on large-scale datasets such as ImageNet, offer a significant advantage in terms of saving time and computational resources. Next, these models are adapted to the Mpox detection task by fine-tuning selected layers in a transfer learning style.
3. **Classification:** The extracted features are passed to fully connected layers to predict a label to indicate whether the image shows signs of Mpox, and the classifier categorizes these predictions into one of the two classes (Mpox or Not Mpox) in the binary classification. In the multiclass classification scenario, the image is classified into one of six categories (e.g., Mpox, Chickenpox, Measles, etc.).
4. **Evaluation and Optimization:** Models are evaluated using validation data and standard metrics. Hyperparameter tuning is employed to enhance model performance and generalization. Detailed experimental settings and metric definitions are explained later in the Experiments and Evaluation section.
5. **Model Deployment:** The best-performing model is selected and deployed within the ITMA’INN mobile application.

#### 3.3.1. Pretrained Models

In this study, we focused primarily on transformer-based and hybrid models, as recent research suggests their superior performance over traditional CNNs in skin lesion classification tasks [13,32]. Hence, we employed a broad range of models, including ViT, TNT, Swin Transformer, MobileViT, ViT Hybrid, and ResNetViT, to evaluate their performance in Mpox detection. Additionally, to establish a strong baseline for comprehensive comparison, we also included the most popular CNN-based models, such as VGG16, ResNet-50, and EfficientNet-B0. With this broad model selection, we could assess the relative strengths of modern transformer-based approaches against standard and well-known CNN architectures. A brief overview of each model and its underlying architecture is provided in the subsequent section.

**Vision Transformer (ViT) [33,34]** **.**

ViT is one of the first transformer architectures designed for computer vision tasks. Inspired by the success of transformers in natural language processing (NLP) [35], several efforts have emerged to apply this success to images. Unlike CNNs, which process images as a whole, ViT divides images into fixed-size patches that serve as tokens similar to the way words are treated in text. These tokens are passed through a standard transformer encoder using self-attention mechanisms to model spatial relationships. ViT relies on data-driven learning, requiring large-scale datasets for training. After the emergence of ViT, several variants have been proposed [36,37,38], with hybrid architectures integrating both characteristics of ViTs and CNNs.

**Transformer in Transformer (TNT) [39]**.

The TNT model extends the standard Vision Transformer by introducing a two-level embedding mechanism that captures local and global representations. Each image is divided into fixed-size patches, which are then further subdivided into a series of pixels. Pixel values are transformed into pixel embeddings via a linear projection to capture the local dependencies. These embeddings are then processed within an inner transformer block. This block’s output is combined to create patch embeddings, which are sent to an outer transformer block that records the global interactions between patches. TNT preserves fine-grained local information while learning high-level global features, enhancing performance in several tasks.

**Swin Transformer [36]**.

Swin Transformer processes images by dividing them into non-overlapping patches, which are passed through hierarchical stages of transformer blocks. A key innovation is the shifted window mechanism, which enables efficient local self-attention while capturing cross-region dependencies. This enables the model to effectively capture relationships between neighboring regions without calculating attention globally. The model constructs a hierarchical architecture, and the number of tokens decreases step by step through patch merging layers, similar to how pooling is done in CNNs. That leaves the multi-scale feature maps, thereby making the Swin Transformer fit for downstream applications like object detection and segmentation. The model’s architecture is scalable in different sizes (e.g., Swin-Tiny, Swin-Base, etc.) and offers linear computational complexity with image size.

**MobileViT [40]**.

MobileViT is a lightweight hybrid architecture that combines the strengths of CNNs and Transformers for mobile-friendly vision tasks. It introduces a novel building block called the MobileViT block, designed to learn both local features (using convolutions) and global representations (using transformers) efficiently. This design enables efficient inference while maintaining high accuracy on resource-constrained devices.

**ViT Hybrid [33]**.

ViT Hybrid extends the standard ViT by using a CNN backbone (e.g., BiT) to extract feature maps, which are then used as input tokens for the transformer encoder. The ViT Hybrid leverages a convolutional backbone, specifically BiT (Big Transfer), to extract feature maps. These convolutional features are used as the initial “tokens” for the transformer encoder. This hybrid approach aims to combine the local spatial inductive bias of CNNs with the global contextual modeling of transformers, resulting in improved learning efficiency and performance on image recognition tasks.

**ResNet - Vision Transformer (ViT) hybrid (ResNetViT) [33]**.

The ResNetViT Hybrid model combines a CNN ResNet with a ViT to effectively capture both local and global features from images. The ResNet backbone extracts spatial feature maps, which are then passed to the ViT encoder for global context modeling using self-attention. This hybrid approach leverages the strengths of CNNs (local detail and inductive bias) and transformers (long-range dependency modeling), resulting in a more powerful and generalizable image representation. The model variant we selected does not include a classification head, so we opted to use it as a feature extractor and appended a custom classification head tailored to our Mpox detection task.

**VGG16 [39]**.

This deep CNN became popular due to its simplicity and good performance on image classification problems. Its structure has 13 convolutional layers and 3 fully connected layers, totaling 16 weight layers (as the name indicates, VGG16). It uses very small 3 × 3 convolution filters, stacked in depth to capture complex patterns. These two aspects combined allow the network to learn hierarchical and rich visual features. After convolution and pooling layers, the network ends with two dense layers of 4096 units each, followed by a final classification layer. ReLU activation is used in all hidden layers, and max pooling reduces the spatial dimensions. While straightforward, VGG16 has become the new benchmark for classification accuracy and has since become a widely used feature extractor in transfer learning across many vision tasks.

**ResNet-50 [41].**

ResNet, or Residual Network, is a deep CNN structure that led to the development of residual learning to make it possible to train very deep networks to be practical and effective. Its key innovation lies in the residual block, which introduced a shortcut (or skip) connection that bypasses one or more layers. Instead of learning the entire output directly, each block learns a residual function (i.e., the difference between the input and the output) that makes the optimization process easier, especially for very deep architectures. This design solves the vanishing gradient issue and the degradation problem (where the deeper models perform less well than the shallower models because of optimization issues). As a result, ResNet models, including ResNet-50 (with 50 layers), can be scaled to hundreds, and thousands of layers, and still successfully train.

**EfficientNet-B0 [42]**.

EfficientNet is a family of convolutional neural networks designed to achieve high accuracy with optimal computational efficiency. It was created with neural architecture search and built predominantly using mobile inverted bottleneck Convolutions (MBConv) blocks, similar to those in MobileNetV2, but enhanced with Squeeze-and-Excitation (SE) blocks for better feature recalibration. A key innovation in EfficientNet is compound scaling, where the model depth, width, and input resolution are all uniformly scaled by constant coefficients. Such a balanced treatment yields significantly better performance than previous models scaling a single dimension. EfficientNet-B0, the baseline model, is lightweight and efficient, with only 5.3 million parameters and 0.39 billion FLOPs, yet it achieves 77.1% top-1 accuracy on ImageNet, outperforming much larger models like ResNet-50.

The feature dimensions extracted from each pretrained model that we have utilized are summarized in Table 5, providing a clear comparison of their output representations and architectural notes. The reported dimensions correspond to the feature extractor layer prior to the classification head. For VGG16, features extracted from the FC2 layer are 4,096 dimensions, although flattening after convolution would yield 25,088 features. The MobileViT-Small and TNT-Small variants used in this study have hidden dimensions of 640 and 384, respectively, while larger variants produce higher-dimensional outputs.

#### 3.3.2. Fine-Tuning and Transfer Learning

To adapt pretrained models to the Mpox image classification task, we applied transfer learning, a popular DL approach that leverages pretrained models to solve new, related problems with limited data. All selected models were initially pretrained on large-scale datasets such as ImageNet-1k or ImageNet-21k, which enabled them to learn strong, general-purpose visual features. We fine-tuned these models by replacing their original classification heads with custom fully connected layers suited to our binary or multiclass classification objectives. To reduce the risk of overfitting and improve training efficiency, we typically froze most of the pretrained layers, particularly the feature extraction blocks, and trained only the new classification head and a few top layers. For transformer-based models such as MobileViT and ViT-Hybrid, we froze the early transformer blocks and fine-tuned only the final transformer layers, including the classification head. Key steps in the Code-level configurations and pipeline included the following:

- Loading the pretrained weights for each model using timm.create_model.
- Freezing most of the backbone layers to preserve general feature representations and fine-tuning the final layers to learn from the Mpox dataset.
- Replacing the classification head with custom fully connected layers for binary or multiclass classification tasks, followed by a Sigmoid activation function for binary classification and a SoftMax activation function for multiclass classification.
- Setting random seeds for reproducibility across multiple experiments.
- Applying classical data augmentation techniques such as resizing, normalization, and interpolation via timm.data.create_transform to improve generalization.
- Using early stopping and other regularization techniques to prevent overfitting based on validation loss.
- Hyperparameter optimization was conducted by tuning several key parameters, with selected tested values summarized in Table 6. Key hyperparameters such as the learning rate, batch size, optimizer, dropout rate, and weight decay were adjusted repeatedly through multiple training iterations.

The hyperparameters were selected through validation-based grid search, with early stopping applied based on validation loss using the specified patience values. No learning rate scheduler was used in our experiments.

## 4. Experiments and Evaluation Protocols

The experimental setup for evaluating the AI models implemented in the ITMA’INN system focused on assessing the performance of multiple pretrained DL models in detecting and classifying Mpox skin lesions from images using several approaches and multiple evaluation metrics.

### 4.1. Experimental Setting

We conducted three main experiments using the datasets independently: a binary classification task (Mpox vs. non-Mpox) based on the MSLD dataset, and two multiclass classification tasks, one using the MSLD v2.0 dataset with six classes (Mpox, Chickenpox, Measles, Cowpox, HFMD, and Healthy skin) and another using the MSID dataset with four classes (Mpox, Chickenpox, Measles, and Normal). All experiments were designed under the same evaluation protocols and employed the same performance metrics to ensure comparability across tasks. For each experiment, we applied two data splitting approaches:

1. Stratified 80:20 train–test split, where each subset included the same proportion of class labels. Importantly, the split was applied at the patient level, ensuring that all images belonging to a single patient (even if they depicted different lesions or body parts) were included entirely in either the training or the test set, thus preventing data leakage.
2. Stratified 5-fold cross-validation (CV), where the data was divided into five folds. In each iteration, one fold was reserved for testing and the remaining four were used for training. This process was also conducted patient-wise whenever identifiers were available, ensuring no overlap of patient images across folds. The results were averaged across all folds to provide a more reliable and generalizable estimate of model performance.

An exception was made for the MSID dataset, where only the 80:20 stratified split was performed to maintain its role as an external dataset without introducing resampling bias. 

In addition to these three primary experiments, we conducted an external validation study to test cross-dataset generalization. In this setting, the best-performing models trained on the six-class MSLD v2.0 dataset were evaluated on the four-class MSID dataset without further fine-tuning. This additional experiment was designed to examine the robustness and transferability of the proposed models to unseen data collected from a different source, thereby providing a stronger indication of their real-world applicability.

### 4.2. Experimental Environment and Implementation Details

All experiments were implemented in Python (v3.10.12) using the PyTorch framework (v2.3.1). To ensure reproducibility, we fixed random seeds (torch.manual_seed(42) and torch.cuda.manual_seed(42)). Additional libraries included Torchvision (v0.18.1) for image transformations, TIMM (v1.0.8) for pretrained transformer architectures, Torchinfo (v1.8.0) for model summaries, scikit-learn (v1.5.1) for evaluation metrics, Matplotlib (v3.9.2) for plotting, Pillow (v10.3.0) for image handling, and TQDM (v4.66.5) for training progress monitoring.

All training and evaluation were performed on Google Colab Pro environments using an NVIDIA Tesla T4 GPU (16 GB VRAM), 2.3 GHz Intel Xeon vCPU, and 12 GB RAM.

### 4.3. Evaluation Metrics

To evaluate the prediction performance of the AI models, several evaluation metrics were calculated. These metrics provide a comprehensive view of the model’s classification performance suited for both binary and multiclass problems. Table 7 summarizes the definitions and corresponding mathematical formulas for each metric. Most of these metrics [43,44] are defined mathematically and derived from the confusion matrix components: true positives (TP), false positives (FP), true negatives (TN), and false negatives (FN). For all performance metrics, higher values (closer to one) indicate better performance, except for the loss metric, where lower values represent better model performance.

## 5. Results and Discussion

In this section, we present and analyze the experimental results obtained using binary and multiclass classification datasets under two evaluation strategies: 80/20 train–test split and 5-fold cross-validation. Several transformer-based and CNN-based pretrained models were assessed and compared. We also performed additional experiments to evaluate model generalization on an independent external dataset. Additional performance analyses, including confusion matrices, ROC curves, and training dynamics of the proposed model, are provided in the Supplementary Materials (Figures S2–S4).

### 5.1. AI Model Prediction Performance

We report the prediction results of the pretrained models across four evaluation tasks: (1) binary classification (Mpox vs. non-Mpox), (2) multiclass classification with six classes, (3) multiclass classification with four classes using the MSID dataset, and (4) cross-dataset external validation, where models trained on MSLD v2.0 were tested on MSID.

#### 5.1.1. Binary Classification Task

We first evaluated the pretrained models on the binary classification dataset (Mpox vs. non-Mpox) to establish a baseline for performance. As shown in Table 8, the highest test accuracy of 97.78% was achieved by VGG16, ViT, TNT, and MobileViT, all demonstrating outstanding overall performance. Three transform-based models (ViT, MobileViT, and TNT) managed to attain high precision and F1-score, supported by robust AUC scores (0.9800), which reflect their high discriminative capabilities. ResNetViT, Swin Transformer, and ViT Hybrid models obtained competitive accuracies of 95.56% and AUCs of 0.9600, reflecting their satisfactory but slightly lower performance compared to the transformer-based models alone. On the other hand, models like ResNet-50 and EfficientNet-B0 attained lower accuracies of 91.11% and 93.33%, respectively, albeit with marginally higher loss values and lower AUCs, indicating that traditional CNN-based models, as good as they were, were trumped by transformer-based or hybrid models in this binary classification task.

Additionally, we applied 5-fold CV using the three best-performing pretrained models to validate their robustness further. While the results were lower compared to the 80:20 train–test split, the models still demonstrated reasonable performance, achieving approximately 89% accuracy and F1-score. This decline in performance can be attributed to the reduced training and testing set sizes per fold, which emphasizes a key limitation of using transformer-based pretrained models on small datasets. These findings suggest that such models benefit significantly from larger datasets and that applying CV settings effectively may require a more extensive collection of labeled Mpox images. Overall, the prediction performance shows that transformer and hybrid transformer models, especially ViT, TNT, and MobileViT, are extremely efficient at Mpox image classification, beating conventional convolutional architectures in most of the evaluation metrics.

#### 5.1.2. Multiclass Classification Task

The same was applied to multiclass classification. Table 9 shows that the ViT Hybrid and ResNetViT models achieved the highest test accuracies of 92.16%, with very high precision, recall, and F1-scores reflecting their superiority in handling multiclass predictions. In particular, ViTHybrid performed best with the lowest loss value of 0.2501 among all the models, while ResNetViT achieved a very high AUC of 0.9823, reflecting good discrimination between multiple classes. EfficientNet-B0 also performed well, with a test accuracy of 89.54% and an extremely high AUC of 0.9797, demonstrating its balance between efficiency and accuracy even in more difficult classification tasks. MobileViT and Swin Transformer followed with accuracies above 84%. ViT, TNT, VGG16, and ResNet-50 lagged, with accuracies between 80.39% and 84.31%, and higher loss values. We can also observe that ViT, TNT, VGG16, and ResNet-50 performed comparatively worse, with accuracies ranging from 80.39% to 84.31% and greater loss values, particularly that of VGG16 (0.7762) and ResNet-50 (0.6292).

These results imply that although transformer-based models are commonly strong candidates for multiclass classification, their performance is influenced by architectural variations and fine-tuning strategies. The findings highlight that hybrid and transformer-based architectures, mainly ViT Hybrid and ResNetViT, consistently outperform conventional CNN-based models in multiclass skin lesions classification tasks.

Across both binary and multiclass experiments, the models demonstrated consistently strong performance. To assess whether these differences were statistically meaningful, we conducted paired *t*-tests on fold-level accuracies obtained from 5-fold cross-validation. The paired *t*-test was selected because it directly compares the same folds across two models, thereby controlling for variability introduced by different train–test splits. In the binary classification task, ViT significantly outperformed VGG16 (*p* = 0.0006 < 0.05), while in the multiclass classification task, ResNetViT showed a statistically significant improvement over ResNet50 (*p* = 0.0063 < 0.01). These findings confirm that transformer-based models provide robust and reliable gains over CNN-based baselines. However, comparisons with previously published works could not be subjected to statistical testing, as prior studies report only single aggregate metrics rather than fold-level distributions. Therefore, our statistical claims are limited to experiments conducted under identical datasets and protocols.

Moreover, to assess real-time feasibility, we measured computational and inference times for MobileViT, ViT Hybrid, and ResNetViT. All models achieved inference speeds well below 20 ms per image (≈50 images per second), confirming their suitability for mobile healthcare deployment. Detailed results, including training time and hardware specifications, are provided in the Supplementary Material (Table S2).

#### 5.1.3. Multiclass Classification Task II and External Validation: MSID

Table 10 shows the performance of the pretrained models on the MSID dataset (4 classes: Monkeypox, Chickenpox, Measles, and Normal). The transformer-based architectures again dominated, with ViT Hybrid achieving the highest accuracy (94.87%), precision (94.96%), F1-score (94.87%), recall (94.87%), and AUC (0.9926). ResNetViT closely followed, reaching an accuracy of 94.23% and an AUC of 0.9913. Other models, such as MobileViT (92.31%) and EfficientNet-B0 (92.31%), also delivered strong results. CNN-based baselines such as VGG16 and ResNet-50 lagged, with accuracies around 85%. These results highlight the robustness of transformer-based models in handling multiclass classification.

To further evaluate generalization across datasets, we tested the best-performing models (ResNetViT and ViT Hybrid) trained on the six-class dataset (MSLD v2.0) directly on the four-class dataset (MSID) without additional fine-tuning. The results summarized in Table 11 show that both models maintained stable performance across classes, achieving overall accuracies of 82% (ResNetViT) and 84% (ViT Hybrid). Both demonstrated high recall for Mpox (0.84 and 0.85, respectively) and Normal skin (0.97 and 0.99), confirming their reliability in detecting the target disease and distinguishing healthy cases. Recall for Chickenpox was lower (0.45 and 0.51), reflecting the inherent difficulty of differentiating visually similar vesicular rashes, a challenge that mirrors real-world diagnostic practice. Nevertheless, the macro and weighted averages remained strong, underscoring the robustness of the models.

These external validation findings demonstrate that the models learned transferable and robust feature representations, performing effectively on independent, unaugmented datasets from different sources. This highlights their potential for reliable real-world clinical deployment beyond the original training distribution.

### 5.2. Ablation Study Results

To evaluate the effect of data augmentation on our top-performing models for both binary and multiclass classification tasks (see Table 12), we conducted an ablation study comparing model performance with and without data augmentation. The results demonstrate that data augmentation is the primary driver of competitive performance, with notable improvements observed across all transformer-based models: MobileViT improved from 89.45% to 92.00% in binary classification, TNT from 95.56% to 97.78%, and ResNetViT from 89.02% to 90.24% in multiclass tasks. 

These findings confirm that data augmentation substantially enhances model robustness and generalization capability, particularly for transformer-based architectures that benefit from increased training data diversity. The improvement is especially notable for models like TNT, which showed the most significant gains, likely due to their complex attention mechanisms being better regularized through augmented training samples.

### 5.3. Comparison with the State-of-the-Art Methods

To evaluate the effectiveness of our proposed system, we benchmarked the best-performing models against prior state-of-the-art methods on both binary and multiclass Mpox classification tasks using the same public dataset versions (MSLD and MSLD v2.0), the same data split strategy (80% training, 20% testing), and the same evaluation metrics.

Table 13 presents a comparison with two studies focused exclusively on binary classification tasks (Mpox vs. Non-Mpox) using the same version of the dataset we utilized in our study (MSLD). These studies include Sahin et al. [28] and Nayak et al. [24], both of which used traditional CNN architectures such as MobileNetV2, GoogLeNet, and ResNet-18. Our MobileViT model outperformed the MobileNetV2-based approach presented by [28], which reported an accuracy of 91.11%. Our MobileViT achieved a remarkably higher accuracy of 97.78%. This represents a 6.67 percentage point improvement in classification accuracy, demonstrating the added value of transformer-based hybrid architectures over traditional CNNs for medical diagnosis tasks like Mpox detection. This result underscores the effectiveness of MobileViT’s hybrid architecture, which integrates local feature learning from convolutions with global context modeling through transformers, a key advancement over the traditional MobileNet.

We also compared our model with the work by Nayak et al. [24] who reported several pretrained model results, but we picked the best two (with the best round results) for direct comparison. GoogLeNet delivered competitive results across most evaluation metrics; however, its recall (sensitivity) was notably lower at 94.12%, indicating that 5.88% of actual Mpox cases were missed. In healthcare context, missing positive cases can be risky, as it may lead to false reassurance, delayed treatment, and increased disease transmission. On the other hand, another pretrained model proposed by the same study [24], ResNet-18, achieved slightly higher results in four evaluation metrics. However, the study lacks details on data splitting protocols, which raises concerns about overfitting. Moreover, it remains a relatively heavier model in terms of structure and computational complexity. In contrast, our use of MobileViT, designed to be lightweight and mobile-friendly, achieved a strong accuracy of 97.78%, with balanced precision and recall. Its hybrid architecture, combining convolutional operations with transformer mechanisms, enabled the effective extraction of both local and global features. Trained on an augmented dataset of 2607 images, MobileViT demonstrated robust generalization and MobileViT and provided an optimal trade-off between accuracy and model efficiency, making it well-suited for deployment in real-time mobile health systems. Its hybrid design, combining convolutional and transformer layers, enabled effective feature extraction of both local and global patterns from skin lesion images, which is crucial for visual diagnosis.

For multiclass classification, Table 14 presents a comparison with the only recent study that applied classification on the same skin lesion dataset (MSLD v2.0), which includes six classes: Mpox, Chickenpox, Measles, Cowpox, HFMD, and Healthy skin [30]. While both studies utilized the same dataset, the methodologies differ notably. Ali and coauthors evaluated several CNN-based models and applied standard and color-space augmentation techniques, achieving their best performance with DenseNet-121 at an accuracy of 83.59%. In contrast, our approach applied a broader set of augmentation techniques and adopted transformer-based architectures, leading to better classification results and stronger generalization by outperforming DenseNet-121 by 8.57% in terms of accuracy. Moreover, the successful classification across six visually similar skin conditions demonstrates the robustness of our system, addressing a diagnostic challenge even for trained clinicians.

Overall, our approach demonstrates the advantages of transfer learning, pretrained model fine-tuning, and careful hyperparameter optimization. By incorporating these strategies, we successfully adapted powerful pretrained models to the domain of skin lesion classification, achieving state-of-the-art outcomes while preserving the computational efficiency needed for clinical and mobile applications.

## 6. Mobile Application and Dashboard Development and Integration

After finalizing and evaluating the AI model pipeline for Mpox detection and to demonstrate the practical use of our model, we moved to the next phase, which is integrating the best-performing, mobile-compatible model into a functional system accessible to end users (i.e., patients). This was done by developing a cross-platform mobile application and an administrative monitoring dashboard.

### 6.1. Mobile Application Design and Testing

We created the ITMA’INN mobile application to provide an intelligent and user-friendly diagnostic tool for the identification of Mpox. The application also allows patients to interact with the system, access relevant features, and receive appropriate guidance and support. The Flutter framework was used to guarantee cross-platform compatibility on both iOS and Android. The app’s backend is hosted on Firebase, leveraging services such as Cloud Functions, Firestore for real-time database operations, and Firebase Authentication for secure user management.

**Figure 3:**
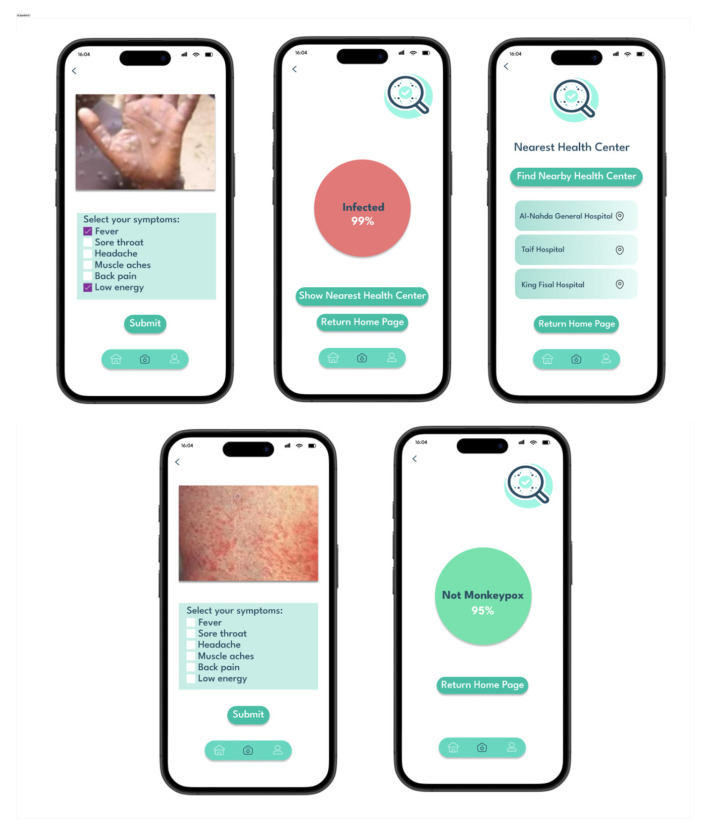
ITMA’INN Application Testing Scenario.

After development, we tested the ITMA’INN app to ensure reliable performance and a user-friendly experience. When users launch the ITMA’INN application for the first time, they are guided through a brief onboarding process before accessing the main diagnostic functionality. The core interaction begins at the Examine Screen, where users submit symptom descriptions and upload skin lesion images from their camera, photo library, or device files, for analysis. Based on the AI model’s prediction, users are directed either to the Infected Screen or the Uninfected Screen. If the user is determined to be infected, the system provides a list of nearby health centers using location services to facilitate prompt medical follow-up. This workflow was tested to ensure reliability in user navigation, accurate result display, and effective health center recommendations based on geolocation data. Figure 3 displays the main user interfaces of the ITMA’INN application, divided into two halves. The top row presents the interaction flow for users predicted to have Mpox. It begins on the Examine Screen, where the user uploads a photo of their skin lesion and selects symptoms. After entering the details, they are taken to the Results Screen, where the AI model prediction and a “Show Nearest Health Center” button are displayed. On clicking this, a screen with a list of health centers near the user’s geographic location is displayed. The bottom row shows the corresponding interaction flow for users who are not predicted to be infected. Such users go through the same first steps, image upload and symptoms selection, but are ultimately taken to a screen saying that no signs of Mpox were detected.

### 6.2. Monitoring Dashboard Design and Testing

Our system includes a comprehensive real-time monitoring dashboard designed specifically for health authorities. The dashboard integrates patient data from the ITMA’INN mobile application through Firebase database, processes it via Google BigQuery, and visualizes it using Power BI with DirectQuery connections for real-time updates. Health authorities access the dashboard through Power BI authentication, requiring organizational credentials, ensuring secure access control. Our system implements several privacy safeguards, including

**Figure 4:**
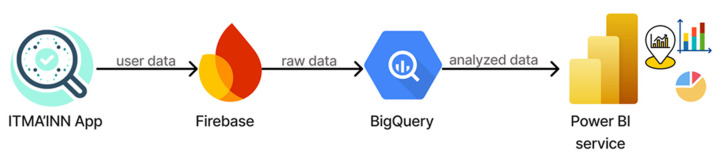
The data flow supporting the Mpox Monitoring Dashboard.

- Secure authentication through organizational credentials;
- Data flows through established enterprise platforms (Firebase, Google BigQuery, Power BI), as shown in Figure 4;
- Session management and credential verification.

The dashboard provides health authorities with essential monitoring capabilities, including infection rates, demographic analysis, and geographical distribution tracking, supporting evidence-based public health decision-making.

**Figure 5:**
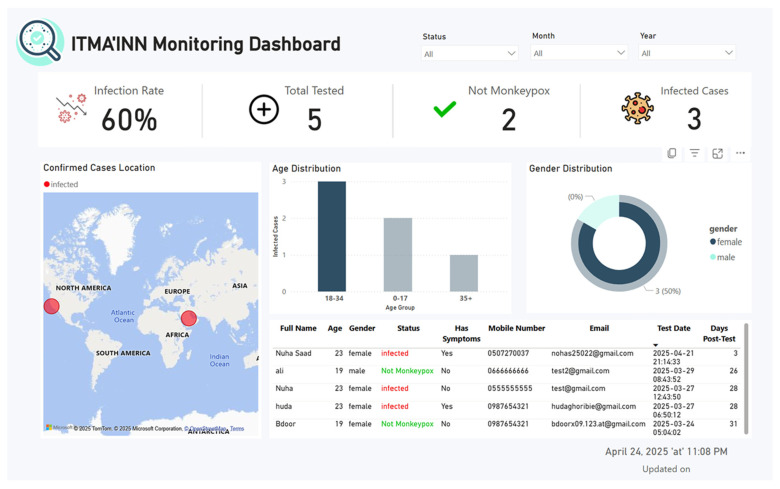
Overview of the monitoring dashboard in the ITMA’INN system.

The dashboard (see Figure 5) provides administrators with a secure, real-time Mpox monitoring system, starting with the Dashboard Login Screen, which requires the organization’s email authentication to access the analytics portal. Once logged in, the Dashboard Overview Screen displays critical metrics like infection rates (e.g., 60%), demographic breakdowns (e.g., 50% female cases), and a detailed patient table with symptoms, test dates, and locations, all updated in real-time. Administrators can track outbreaks, analyze trends, and make data-driven decisions, while the system ensures security through strict credential checks and session management. More figures of ITMA’INN Monitoring Dashboard are provided in the Supplementary Materials (Figures: S5–S7). Testing focuses on login validation, data accuracy, real-time updates, and responsiveness, ensuring reliable performance for public health monitoring.

## 7. Study Limitations

Despite the strengths of this study, including the use of multiple publicly available datasets, robust evaluation across binary and multiclass tasks, external validation to test generalization, and the potential for real-time deployment in smart healthcare settings, the work still suffers from several limitations.

First, the reliance on publicly available datasets without validation on real patient data from clinical settings. While our models demonstrated strong performance across multiple benchmark datasets and external validation experiments, the transition from research datasets to real-world clinical applications requires additional validation steps. Challenges in accessing real patient data include institutional review board approvals, patient consent requirements, healthcare data privacy regulations, and the sporadic nature of Mpox outbreaks. Future clinical validation studies should involve prospective data collection in partnership with dermatology departments and infectious disease units, comparative analysis against dermatologist diagnoses, and multi-site validation across diverse patient populations. Until such validation is completed, our system should be considered a decision-support tool rather than a standalone diagnostic solution.

Second, while pretrained transformer-based and CNN-based models achieved high accuracy, they also present inherent architectural limitations. Since these models were trained on natural images (e.g., ImageNet), they may not fully capture the domain-specific features of skin lesions. Larger architectures, such as ResNetViT and Transformer-in-Transformer, resulted in longer inference times, potentially affecting their suitability for real-time mobile deployment. Some models were also sensitive to class imbalance in multiclass settings, and ViT-based models required larger datasets to generalize effectively. These issues highlight trade-offs in speed, dataset dependency, and robustness.

Finally, our framework did not incorporate interpretability using XAI methods such as LIME or Grad-CAM, which are essential for transparency and clinical trust in AI systems. Future work should integrate explainable AI techniques to verify that the models focus on diagnostically relevant lesion regions and not on background artifacts.

Together, these limitations highlight the need for further refinement through explainability, prospective clinical validation, and optimization for real-time mobile deployment.

## 8. Conclusions

This paper proposed the design and development of ITMA’INN, an intelligent, AI-powered mobile healthcare system for the early detection and monitoring of Mpox from skin lesion images by integrating deep learning pretrained models into a user-friendly mobile application. It relies on the Mobile Vision Transformer (MobileViT) model and integrates the mobile application with a dashboard for healthcare authorities. This combination has made the system a practical application in healthcare environments, achieving high accuracy in binary classification up to 97.8% and 90% in multi-class classification, thus outperforming traditional CNN-based systems. The key advantages of the system are as follows:

- Lightweight transformer architecture optimized for smartphone deployment, enabling widespread community access for early detection;
- Real-time monitoring dashboard providing continuous epidemiological surveillance and trend analysis for health authorities;
- The system reduces the pressure on hospitals for early detection, which contributes to limiting infection transmission and improving healthcare quality;
- Scalable architecture capable of expansion to other dermatological conditions.

In this way, ITMA’INN forms a comprehensive and innovative system, combining scientific effectiveness with practical application, emphasizing its importance in enhancing healthcare systems, supporting public health, and responding to epidemics effectively.

For future enhancement, we aim to expand the scope of diagnosis to include other skin diseases using larger and more diverse datasets, which will contribute to early detection and improved diagnostic accuracy. We also plan to enhance multiclass classification performance through additional model training and fine-tuning to support broader diagnostic capabilities. At the same time, we recognize the importance of validating the monitoring dashboard through formal collaborations with health authorities and clinical institutions. Future studies will therefore involve pilot testing of the dashboard in healthcare settings, development of formal privacy and data protection policies, and ensuring compliance with relevant regulatory frameworks. We also aim to establish clinical partnerships and pursue comparative studies against dermatologist diagnoses across multiple healthcare settings.

By implementing these future advancements, ITMA’INN system will be enhanced, contributing not only to the early detection of Mpox but also to the broader prevention of other infectious diseases.

## Figures and Tables

**Table 1:** Comparative analysis of recent Mpox detection studies.

Study (Year)	Task	Dataset	Split	Metrics	Classification Used	Limitations	Best Model
Nayak et al. (2023) [24]	Binary (Mpox vs. non-Mpox)	Public Mpox dataset (skin lesion images)	Not specified (train/test)	Accuracy, Precision, Recall, F1	CNNs (ResNet-18, GoogLeNet, SqueezeNet, AlexNet)	Lack of detail on splitting protocol; limited dataset size	ResNet-18 (99.49% accuracy)
Altun et al. (2023) [25]	Binary	Public skin lesion dataset	80/20, hyperparameter tuning	Accuracy, F1, Recall, AUC	CNNs (MobileNetV3-s, EfficientNetV2, ResNet-50, VGG19, DenseNet-11)	Possible overfitting due to limited diversity; needs real-world testing	MobileNetV3-s (96% accuracy, F1 = 0.98, AUC = 0.9)
Jaradat et al. (2023) [26]	Binary	Public dataset (skin lesion images)	Train/test (fine-tuned)	Accuracy, Recall, Precision, F1	CNNs (MobileNetV2, VGG19, VGG16, ResNet-50, EfficientNet3)	Dataset imbalance; limited validation in clinical practice	MobileNetV2 (98.16% accuracy, F1 = 0.9)
Thieme et al. (2023, Nature Medicine) [27]	Binary & Multiclass	Large clinical + dermoscopic dataset (~130k images)	Cross-validation	Accuracy	CNN-based (ResNet-18, ResNet-34, DenseNet-19, VGG19_b)	High computational cost; dataset may not generalize across populations	MPXV-CNN (90% accuracy, robust real-world deployment)
Ali et al. (2022) [29]	Multiclass (6 classes)	MSLD v2.0 (Kaggle)	Train/test	Accuracy	CNNs (DenseNet-121, VGG, ResNet)	Moderate accuracy; limited augmentation	DenseNet-121 (83.59% accuracy)

**Table 2:** Distribution of original and augmented images per class in the binary classification dataset (MSLD).

Image Classes	Original Images	Augmented Images (Training Only)
Mpox	102	1148
Non-Mpox	126	1414
Total	228	2562

**Table 3:** Distribution of original and augmented images per class in the multiclass classification dataset (MSLD v2.0).

Image Classes	Original Images	Augmented Images (Training Only)
Mpox	284	2579
Chickenpox	75	725
Measles	55	565
Cowpox	66	662
HFMD	161	1546
Healthy	114	1131
Total	755	7208

**Table 4:** Original images per class in the benchmark dataset (MSID).

Image Classes	Original Images
Mpox	279
Chickenpox	107
Measles	91
Healthy	293
Total	770

**Table 5:** Feature dimensions of the output layer from each pretrained model used as a feature extractor.

Model	Variant Used	Feature Dimension	Key Architectural Note
VGG16	Standard (VGG16)	4096	Features extracted from the second fully connected layer (FC2)
ResNet-50	Standard (ResNet50)	2048	Features extracted after global average pooling
EfficientNet-B0	B0	1280	Features from the final embedding layer before classification
Vision Transformer (ViT)	Base/16 (vit_b_16)	768	Hidden size of the transformer encoder
Transformer-iN-Transformer (TNT)	Small (tnt_s_patch16_224)	384	Hidden size of the transformer encoder
Swin Transformer	Tiny (swin_t)	768	Hidden size of the transformer encoder
MobileViT	Small (mobilevit_s)	640	Embedding size from the MobileViT block
ViT Hybrid	Base-BiT-384 (vit-hybrid-base)	768	Hidden size of the hybrid transformer encoder
ResNetViT	vit_base_r50_s16_224	768	ResNet-50 backbone with ViT encoder; hidden size is 768

**Table 6:** Tested hyperparameter ranges with selected values in bold used for final model evaluation.

Hyperparameters	Values
Learning rate	**0.001**, 0.0001, 1 × 10^−5^, 2 × 10^−5^
Batch size	8, **16**, 32
Dropout rate	0.2, **0.3**, 0.4
Weight decay	1 × 10^−4^, 1 × 10^−5^
Optimizer	**Adam**, AdamW
Epochs	**5**, **10**, 50, 100
Early stopping	**3**, 5, **10**, 15, 50

**Table 7:** Performance Evaluation Metrics of the DL pretrained models.

Metrics	Definition	Mathematical Formula
Accuracy (Acc)	It is the ratio of correctly classified samples to the total number of samples in the dataset.	Acc=(TP+TN)/(TP+TN+FP+FN)
Precision (PR)	The proportion of true positive predictions among all positive predictions, reflecting the model’s ability to avoid false positives.	PR=TP/(TP+FP)
Recall (RC)	The proportion of true positives among all actual positives, indicating how well the model captures relevant cases.	RC=TP/(TP+FN)
F1-score	The harmonic mean of precision and recall, providing a balance between the two.	*F1-score* = (2·PR·RC)/(PR+RC)
AUC-ROC	Is the area under the ROC curve, measuring the model’s ability to distinguish between classes	*AUC* $=\sum_{i=1}^{n-1}\left(FPR_{i+1}-FPR_{i}\right)\cdot \frac{TPR_{i+1}+TPR_{i}}{2}$
Loss	It measures how well the model’s predictions match the actual outcomes, representing the model’s prediction error.	*MSE =* $\frac{1}{N}\sum_{i=1}^{N}\left(y_{i}-\hat{y_{i}}\right)$

**Table 8:** Prediction Performance of Several Pretrained Models on the Binary Classification Task. **Bold and underlined** font indicates the best-performing models, and **Bold** font indicates the second best-performing models.

Model Name	Accuracy	Precision	F1-Score	Recall	Loss	AUC
80:20 Train–Test Split						
ResNetViT	**0.9556**	**0.9596**	**0.9557**	**0.9556**	**0.2355**	**0.9600**
ResNet-50	0.9111	0.9152	0.9114	0.9111	0.2575	0.9150
TNT	**0.9778**	**0.9788**	**0.9778**	**0.9778**	**0.1729 **	**0.9800**
Swin Transformer	0.9556	0.9596	0.9557	0.9556	0.1770	0.9600
ViT	**0.9778**	**0.9788**	**0.9778**	**0.9778**	**0.1669**	**0.9800**
VGG16	**0.9778**	**0.9788**	**0.9778**	**0.9778**	**0.1030**	**0.9800**
ViT Hybrid	0.9556	0.9596	0.9557	0.9556	0.1656	0.9600
MobileViT	**0.9778**	**0.9786**	**0.9777**	**0.9788**	**0.1967**	**0.9750**
EfficientNet-B0	0.9333	0.9345	0.9335	0.9333	0.2969	0.9720
5-Fold Cross-validation						
ViT	**0.8903**	**0.8914**	**0.8898**	**0.8903**	**0.4701**	**0.8871**
MobileViT	0.8773	0.8783	0.8768	0.8773	0.4133	0.8731
TNT	**0.8990**	**0.9022**	**0.8984**	**0.8990**	**0.5395**	**0.8944**
ResNet-50	0.7971	0.8050	0.7914	0.7971	0.6498	0.7808
VGG16	0.7783	0.7809	0.7744	0.7783	0.8790	0.7648

**Table 9:** Prediction Performance of Several Pretrained Models on the Multiclass Skin Lesion Classification Task. **Bold and Underlined** font indicates the best-performing models and **Bold** font indicates the second best-performing model.

Model Name	Accuracy	Precision	F1-Score	Recall	Loss	AUC
80:20 Train–Test Split						
ResNetViT	**0.9216**	**0.9271**	**0.9224**	**0.9216**	**0.3079**	**0.9823**
ResNet-50	0.8039	0.8169	0.8043	0.8039	0.6292	0.9509
TNT	0.8170	0.8301	0.8157	0.8170	0.4797	0.9611
Swin Transformer	0.8497	0.8596	0.8503	0.8497	0.4288	0.9736
ViT	0.8431	0.8551	0.8413	0.8431	0.5175	0.9655
VGG16	0.8039	0.8062	0.8046	0.8039	0.7762	0.9570
ViT Hybrid	**0.9216**	**0.9262**	**0.9219**	**0.9216**	**0.2501**	**0.9286**
MobileViT	0.8758	0.8782	0.8746	0.8758	0.4905	0.9730
EfficientNet-B0	**0.8954**	**0.9002**	**0.8961**	**0.8954**	**0.3559**	**0.9797**
5-Fold Cross-validation						
ResNetViT	**0.85288**	**0.85734**	**0.85188**	**0.85288**	**0.45666**	**0.9665**
ViT Hybrid	**0.8388**	**0.84282**	**0.83604**	**0.8388**	**0.45642**	**0.96602**
ViT	0.7814	0.7824	0.7771	0.7814	0.6157	0.9450
ResNet-50	0.7572	0.7643	0.7535	0.7572	0.6866	0.9327
VGG16	0.7145	0.7209	0.7105	0.7145	1.0992	0.9080

**Table 10:** Prediction Performance of Several Pretrained Models on external 4-class Validation with Benchmark Dataset. Bold and underlined font indicates the best-performing models, and bold font indicates the second best-performing model.

Model Name	Accuracy	Precision	F1-Score	Recall	Loss	AUC
80:20 Train–Test Split						
ResNetViT	**0.9423**	**0.9427**	**0.9423**	**0.9423**	**0.9913**	**0.9423**
ResNet-50	0.8590	0.8663	0.8561	0.8590	0.9687	0.8590
TNT	0.8526	0.8487	0.8493	0.8526	0.9717	0.8526
Swin Transformer	0.8846	0.8871	0.8849	0.8846	0.9729	0.8846
ViT	0.8846	0.8884	0.8848	0.8846	0.9708	0.8846
VGG16	0.8526	0.8512	0.8487	0.8526	0.9682	0.8526
ViT Hybrid	**0.9487**	**0.9496**	**0.9487**	**0.9487**	**0.9926**	**0.9487**
MobileViT	0.9231	0.9255	0.9240	0.9231	0.9785	0.9231
EfficientNet-B0	**0.9231**	**0.9231**	**0.9224**	**0.9231**	**0.9812**	**0.9231**

**Table 11:** Cross-dataset classification performance of ResNetViT and ViT Hybrid trained on MSLD v2.0 (6 classes) and tested on the independent MSID dataset (4 classes).

Model	Accuracy	Precision (Macro)	Recall (Macro)	F1-Score (Macro)	Precision (Weighted)	Recall (Weighted)	F1-Score (Weighted)
ResNetViT	0.82	0.84	0.75	0.78	0.83	0.82	0.81
ViT Hybrid	0.84	0.84	0.76	0.79	0.84	0.84	0.83

**Table 12:** Ablation study evaluating the impact of data augmentation on the top-performing models for both multiclass and binary classification tasks. Bold values highlight the best results for each model with augmentation.

Classification Task	Model	Accuracy	Precision	F1-Score	Recall	AUC
Multiclass Classification (6 Classes)	ResnetViT	0.8902	0.8913	0.888	0.890	0.9718
	**ResnetViT + Data Augmentation**	**0.9024**	**0.9177**	**0.9065**	**0.902**	**0.9808**
	ViT Hybrid	0.8659	0.8719	0.8666	0.865	0.9708
	**ViT Hybrid + Data Augmentation**	**0.8720**	**0.8804**	**0.8731**	**0.872**	**0.9734**
Binary Classification	MobileViT	0.8667	0.8667	0.8667	0.866	0.8650
	**MobileViT +Data Augmentation**	**0.9778**	**0.9786**	**0.9777**	**0.978**	**0.9750**
	TNT	0.9556	0.9596	0.9557	0.955	0.9600
	**TNT + Data Augmentation**	**0.9778**	**0.9788**	**0.9778**	**0.977**	**0.9800**

**Table 13:** Comparison with Binary Classification Studies Using the MSLD Dataset. **Bold and Underlined** font indicates the best-performing models, and **Bold** font indicates the second best-performing model.

Study	Model	Accuracy (%)	Precision (%)	Recall (%)	F1-Score (%)	AUC (%)
Sahin et al. [28]	MobileNetv2	91.11%	90.00%	90.00%	90.00%	-
Nayak et al. [24]	ResNet-18	**99.49%**	**98.52%**	**99.44%**	**99.49%**	**-**
	GoogLeNet	97.37%	97.46%	94.12%	97.05%	-
Proposed system: ITMA’INN	MobileViT	**97.77%**	**97.86%**	**97.88%**	**97.77%**	**97.50%**

**Table 14:** Comparison with Multiclass Classification Studies Using the MSLDv2.0 Dataset. **Bold** font indicates the best-performing models.

Study	Model	Accuracy (%)	Precision (%)	Recall (%)	F1-Score (%)	AUC (%)
Ali et al. [30]	DenseNet-121	83.59%	85%	-	-	-
Proposed system: ITMA’INN	**ResNetViT**	**92.16%**	**92.71%**	**92.16%**	**92.24%**	**98.23%**

## Data Availability

All datasets employed in both the binary and multiclass classification tasks are publicly accessible via the Kaggle repository. The corresponding links are as follows: (1) MSLD Binary Dataset: https://www.kaggle.com/datasets/nafin59/monkeypox-skin-lesion-dataset (accessed on 15 January 2025); (2) MSLD v2.0 Dataset (6 Classes): https://www.kaggle.com/datasets/joydippaul/mpox-skin-lesion-dataset-version-20-msld-v20 (accessed on 1 March 2025); (3) Monkeypox Skin Lesion Dataset (MSLD, 4 Classes): https://www.kaggle.com/datasets/dipuiucse/monkeypoxskinimagedataset (accessed on 10 September 2025);. The implementation of our proposed **MobileViT-based model** is publicly available on GitHub: https://github.com/MahaThafar/Mpox-Skin-Lesion-Detection-and-Real-Time-Monitoring-in-a-Smart-Healthcare-System/tree/main.

## Supplementary Materials

The following supporting information can be downloaded at https://www.mdpi.com/article/10.3390/diagnostics15192505/s1, Figure S1. Loss, Accuracy and Recall graphs of the proposed model. Figure S2: Precision, F1-Score and AUC graphs of the proposed model. Figure S3: Confusion matrix and ROC Curve graphs of the proposed model. Figure S4: Portal interface of the ITMA’INN system showing the entry point to the real-time monitoring dashboard. Figure S5: Sample view of the ITMA’INN monitoring dashboard displaying infection rate, demographic distributions, case locations, and patient details in an interactive format. Figure S6: Extended view of the ITMA’INN monitoring dashboard, illustrating data filtered by gender. Table S1: Dataset summary and characteristics. Table S2: Computational cost and inference time of the proposed MobileViT binary model and Multiclass models.

## References

[1] Sandoiu A. Why Does SARS-CoV-2 Spread So Easily?. https://www.medicalnewstoday.com/articles/why-does-sars-cov-2-spread-so-easily.

[2] Multi-Country Monkeypox Outbreak in Non-Endemic Countries: Update. https://www.who.int/emergencies/disease-outbreak-news/item/2022-DON388.

[3] Sorayaie Azar A., Naemi A., Babaei Rikan S., Bagherzadeh Mohasefi J., Pirnejad H., Wiil U.K. (2023). Monkeypox Detection Using Deep Neural Networks. BMC Infect. Dis..

[4] Nasr M.M., Islam M.M., Shehata S., Karray F., Quintana Y. (2021). Smart Healthcare in the Age of AI: Recent Advances, Challenges, and Future Prospects. IEEE Access.

[5] Albaradei S., Thafar M.A., Alsaedi A., Van Neste C., Gojobori T., Essack M., Gao X. (2021). Machine Learning and Deep Learning Methods That Use Omics Data for Metastasis Prediction. Comput. Struct. Biotechnol. J..

[6] Thafar M.A., Albaradei S., Uludag M., Alshahrani M., Gojobori T., Essack M., Gao X. (2023). OncoRTT: Predicting Novel Oncology-Related Therapeutic Targets Using BERT Embeddings and Omics Features. Front. Genet..

[7] Alamro H., Thafar M.A., Albaradei S., Gojobori T., Essack M., Gao X. (2023). Exploiting Machine Learning Models to Identify Novel Alzheimer’s Disease Biomarkers and Potential Targets. Sci. Rep..

[8] Zhang P., Boulos M. (2023). Generative AI in Medicine and Healthcare: Promises, Opportunities and Challenges. Future Internet.

[9] Albaradei S., Alganmi N., Albaradie A., Alharbi E., Motwalli O., Thafar M.A., Gojobori T., Essack M., Gao X. (2023). A Deep Learning Model Predicts the Presence of Diverse Cancer Types Using Circulating Tumor Cells. Sci. Rep..

[10] Tsuneki M. (2022). Deep Learning Models in Medical Image Analysis. J. Oral Biosci..

[11] Li X., Zhang L., Yang J., Teng F. (2024). Role of Artificial Intelligence in Medical Image Analysis: A Review of Current Trends and Future Directions. J. Med. Biol. Eng..

[12] De A., Mishra N., Chang H.-T. (2024). An Approach to the Dermatological Classification of Histopathological Skin Images Using a Hybridized CNN-DenseNet Model. PeerJ Comput. Sci..

[13] Yolcu Oztel G. (2024). Vision Transformer and CNN-Based Skin Lesion Analysis: Classification of Monkeypox. Multimed. Tools Appl..

[14] Curti N., Merli Y., Zengarini C., Giampieri E., Merlotti A., Dall’Olio D., Marcelli E., Bianchi T., Castellani G. (2022). Effectiveness of Semi-Supervised Active Learning in Automated Wound Image Segmentation. Int. J. Mol. Sci..

[15] Griffa D., Natale A., Merli Y., Starace M., Curti N., Mussi M., Castellani G., Melandri D., Piraccini B.M., Zengarini C. (2024). Artificial Intelligence in Wound Care: A Narrative Review of the Currently Available Mobile Apps for Automatic Ulcer Segmentation. BioMedInformatics.

[16] Fiscone C., Curti N., Ceccarelli M., Remondini D., Testa C., Lodi R., Tonon C., Manners D.N., Castellani G. (2024). Generalizing the Enhanced-Deep-Super-Resolution Neural Network to Brain MR Images: A Retrospective Study on the Cam-CAN Dataset. eNeuro.

[17] Musthafa M.M., R M.T., V V.K., Guluwadi S. (2024). Enhanced Skin Cancer Diagnosis Using Optimized CNN Architecture and Checkpoints for Automated Dermatological Lesion Classification. BMC Med. Imaging.

[18] Debelee T.G. (2023). Skin Lesion Classification and Detection Using Machine Learning Techniques: A Systematic Review. Diagnostics.

[19] Singh R.K., Gorantla R., Allada S.G.R., Narra P. (2022). SkiNet: A Deep Learning Framework for Skin Lesion Diagnosis with Uncertainty Estimation and Explainability. PLoS ONE.

[20] Sreekala K., Rajkumar N., Sugumar R., Sagar K.V.D., Shobarani R., Krishnamoorthy K.P., Saini A.K., Palivela H., Yeshitla A. (2022). Skin Diseases Classification Using Hybrid AI Based Localization Approach. Comput. Intell. Neurosci..

[21] Chadaga K., Prabhu S., Sampathila N., Nireshwalya S., Katta S.S., Tan R.-S., Acharya U.R. (2023). Application of Artificial Intelligence Techniques for Monkeypox: A Systematic Review. Diagnostics.

[22] Patel M., Surti M., Adnan M. (2023). Artificial Intelligence (AI) in Monkeypox Infection Prevention. J. Biomol. Struct. Dyn..

[23] Asif S., Zhao M., Li Y., Tang F., Ur Rehman Khan S., Zhu Y. (2024). AI-Based Approaches for the Diagnosis of Mpox: Challenges and Future Prospects. Arch. Comput. Methods Eng..

[24] Nayak T., Chadaga K., Sampathila N., Mayrose H., Gokulkrishnan N., G M.B., Prabhu S., S S.K., Umakanth S. (2023). Deep Learning Based Detection of Monkeypox Virus Using Skin Lesion Images. Med. Nov. Technol. Devices.

[25] Altun M., Gürüler H., Özkaraca O., Khan F., Khan J., Lee Y. (2023). Monkeypox Detection Using CNN with Transfer Learning. Sensors.

[26] Jaradat A.S., Al Mamlook R.E., Almakayeel N., Alharbe N., Almuflih A.S., Nasayreh A., Gharaibeh H., Gharaibeh M., Gharaibeh A., Bzizi H. (2023). Automated Monkeypox Skin Lesion Detection Using Deep Learning and Transfer Learning Techniques. Int. J. Environ. Res. Public Health.

[27] Thieme A.H., Zheng Y., Machiraju G., Sadee C., Mittermaier M., Gertler M., Salinas J.L., Srinivasan K., Gyawali P., Carrillo-Perez F. (2023). A Deep-Learning Algorithm to Classify Skin Lesions from Mpox Virus Infection. Nat. Med..

[28] Sahin V.H., Oztel I., Yolcu Oztel G. (2022). Human Monkeypox Classification from Skin Lesion Images with Deep Pre-Trained Network Using Mobile Application. J. Med. Syst..

[29] Ali S.N., Ahmed M.T., Paul J., Jahan T., Sani S.M.S., Noor N., Hasan T. (2022). Monkeypox Skin Lesion Detection Using Deep Learning Models: A Feasibility Study. arXiv.

[30] Ali S.N., Ahmed M.T., Jahan T., Paul J., Sani S.M.S., Noor N., Asma A.N., Hasan T. (2024). A Web-Based Mpox Skin Lesion Detection System Using State-of-the-Art Deep Learning Models Considering Racial Diversity. Biomed. Signal Process. Control.

[31] Bala D., Hossain M.S., Hossain M.A., Abdullah M.I., Rahman M.M., Manavalan B., Gu N., Islam M.S., Huang Z. (2023). MonkeyNet: A robust deep convolutional neural network for monkeypox disease detection and classification. Neural Netw..

[32] Nie Y., Sommella P., Carratù M., O’Nils M., Lundgren J. (2022). A Deep CNN Transformer Hybrid Model for Skin Lesion Classification of Dermoscopic Images Using Focal Loss. Diagnostics.

[33] Dosovitskiy A., Beyer L., Kolesnikov A., Weissenborn D., Zhai X., Unterthiner T., Dehghani M., Minderer M., Heigold G., Gelly S. (2020). An Image Is Worth 16x16 Words: Transformers for Image Recognition at Scale. arXiv.

[34] Mao X., Qi G., Chen Y., Li X., Duan R., Ye S., He Y., Xue H. Towards Robust Vision Transformer. Proceedings of the 2022 IEEE/CVF Conference on Computer Vision and Pattern Recognition (CVPR).

[35] Vaswani A., Shazeer N.M., Parmar N., Uszkoreit J., Jones L., Gomez A.N., Kaiser L., Polosukhin I. Attention Is All You Need. Proceedings of the 31st Conference on Neural Information Processing Systems (NIPS 2017).

[36] Liu Z., Lin Y., Cao Y., Hu H., Wei Y., Zhang Z., Lin S., Guo B. Swin Transformer: Hierarchical Vision Transformer Using Shifted Windows. Proceedings of the 2021 IEEE/CVF International Conference on Computer Vision (ICCV).

[37] Zhou D., Kang B., Jin X., Yang L., Lian X., Jiang Z., Hou Q., Feng J. (2021). DeepViT: Towards Deeper Vision Transformer. arXiv.

[38] Han K., Wang Y., Chen H., Chen X., Guo J., Liu Z., Tang Y., Xiao A., Xu C., Xu Y. (2023). A Survey on Vision Transformer. IEEE Trans. Pattern Anal. Mach. Intell..

[39] Simonyan K., Zisserman A. (2014). Very Deep Convolutional Networks for Large-Scale Image Recognition. arXiv.

[40] Mehta S., Rastegari M. (2021). MobileViT: Light-Weight, General-Purpose, and Mobile-Friendly Vision Transformer. arXiv.

[41] He K., Zhang X., Ren S., Sun J. Deep Residual Learning for Image Recognition. Proceedings of the 2016 IEEE Conference on Computer Vision and Pattern Recognition (CVPR).

[42] Tan M., Le Q.V. (2019). EfficientNet: Rethinking Model Scaling for Convolutional Neural Networks. arXiv.

[43] Davis J., Goadrich M. The Relationship between Precision-Recall and ROC Curves. Proceedings of the 23rd International Conference on Machine Learning—ICML ’06.

[44] Martinez-Ramon M., Ajith M., Kurup A.R. (2024). Deep Learning: A Practical Introduction.

