# Supplementary material for: Deep Learning-Based Mpox Skin Lesion Detection and Real-Time Monitoring in a Smart Healthcare System
Source: Diagnostics (Basel). 2025 Oct 1;15(19):2505. doi: 10.3390/diagnostics15192505 (PMC12523520; doi:10.3390/diagnostics15192505)
Supplement: Supplementary file 1 [file diagnostics-15-02505-s001.zip › diagnostics-3828092-supplementary.pdf]

Deep Learning-Based Monkeypox Skin Lesion Detection and Real-Time Monitoring in a Smart Healthcare System.

SUPPLEMENTARY MATERIAL

Huda Alghoraibi [1], Nuha Alqurashi [1], Sarah Alotaibi [1], Renad Alkhudaydi [1], Bdoor Aldajani [1], Jood Batweel [1], Lubna Alqurashi [1], Azza Althagafi[1], and Maha A. Thafar [1,*]

[1] Department of Computer Science, College of Computers and Information Technology, Taif University, Taif, Saudi Arabia.

*Correspondence: (e-mail: m.thafar@tu.edu.sa)

Supplementary Tables and Figures

1. Dataset Description

A compact dataset summary (**dataset**/version & license, classes, #images & #patients, modality, patient-wise split details, augmentations, caveats) is provided in Table S1.

Table S1. Dataset summary and characteristics.

Dataset / Version	License	Classes	Images	Patients	Modality	Augmentations	Caveats
**Monkeypox Skin Lesion Dataset (MLSD)**[25]	Public	2 (Monkeypox, Non-Monkeypox: Chickenpox + Measles)	228 original 2562 augmented (training only)	Binary 20/80: patient-wise Binary 5CV: not patient-wise	Skin images	Rotation, translation, reflection, shear, color adjustment, noise, sharpening, blurring, elastic deformation, brightness, scaling	Small dataset; 5CV split not patient-wise, may cause data leakage
**Mpox Skin Lesion Dataset v2.0 (MSLD v2.0)** [26]	Public	6 (Monkeypox, Chickenpox, Measles, Cowpox, HFMD, Healthy)	755 original 6653 augmented (training only)	Multi 20/80: not patient-wise Multi 5CV: patient-wise	Skin images	Same augmentations as above	Multi 20/80 split not patient-wise, potential optimistic performance if images from same patient appear in both train/test
**Monkeypox Skin Images Dataset (MSID)**[27]	Public	4 (Monkeypox,Chickenpox,Measles,Normal)	770 original	Multi 20/80: not patient-wise	Skin images	None	The 80/20 split is not patient-wise. Relatively small dataset (770 images only). Class

							imbalance

2. Computational and Inference Time Analysis

To evaluate real-time feasibility, we measured training time and inference time per image for the proposed MobileViT model (binary classification) and two representative multiclass baselines (ViT Hybrid and ResNetViT). Across all models, inference time remained below 20 ms per image (~50 images per second), confirming that MobileViT and the baselines are suitable for real-time deployment in mobile healthcare applications and clinical decision-support systems.

Table S2. Computational cost and inference time of the proposed MobileViT binary model and Multiclass models.

Model	Setting	Total Training Time (s)	Avg. Training Time per Epoch (s)	Inference Time (ms)
**MobileViT**	Binary Classification	58.88	11.78	8.60
**ViT Hybrid**	Multiclass Classification	3272.84	297.53	15.73
**ResNetViT**	Multiclass Classification	1863.14	116.44	14.58

3. Model Performance Analysis

Additional performance analyses, including confusion matrices, ROC curves, and training dynamics of the proposed model, are provided in the Supplementary Materials (Figures S1–S6).

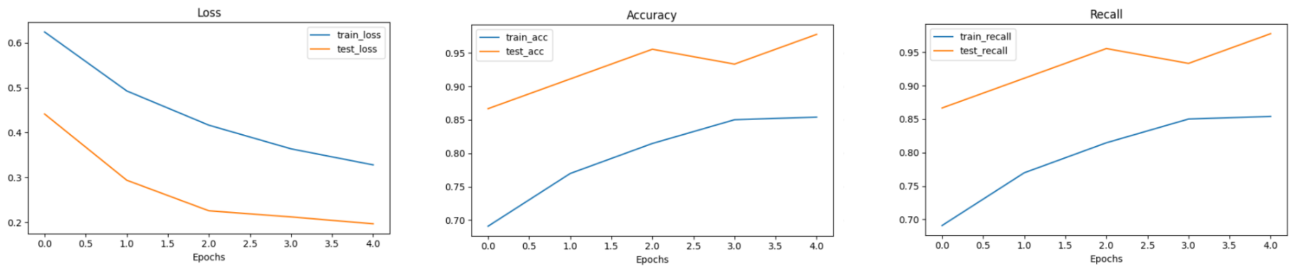

Figure S1. Loss, Accuracy and Recall graphs of the proposed model.

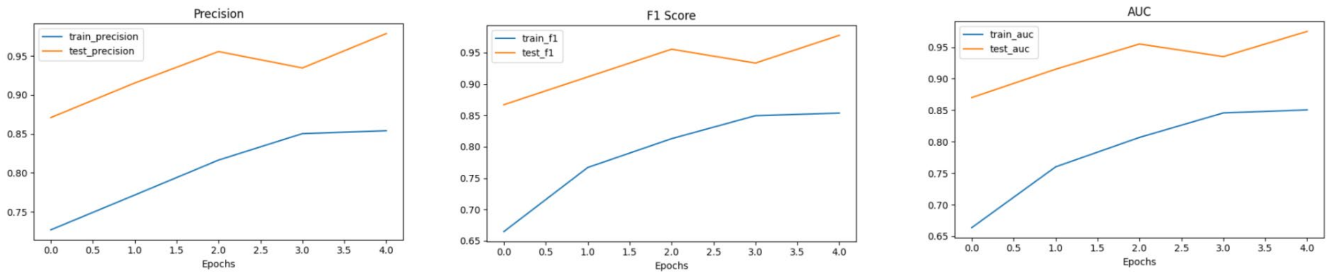

Figure S2. Precision, F1-Score and AUC graphs of the proposed model.

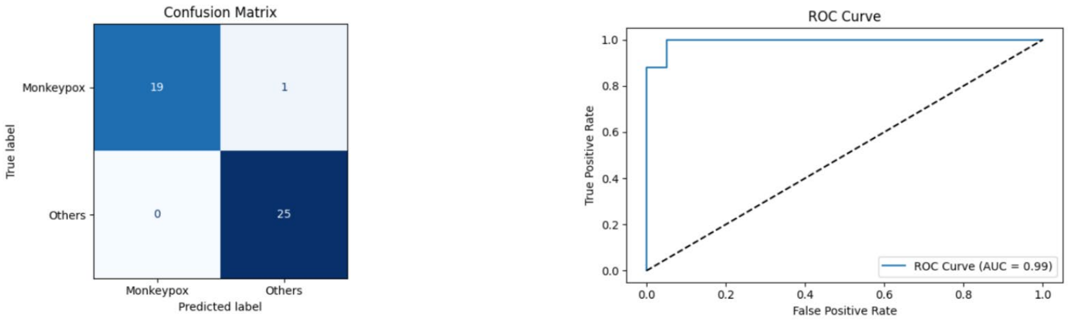

Figure S3. Confusion matrix and ROC Curve graphs of the proposed model.

4. Different Screenshots of the Dashboard.

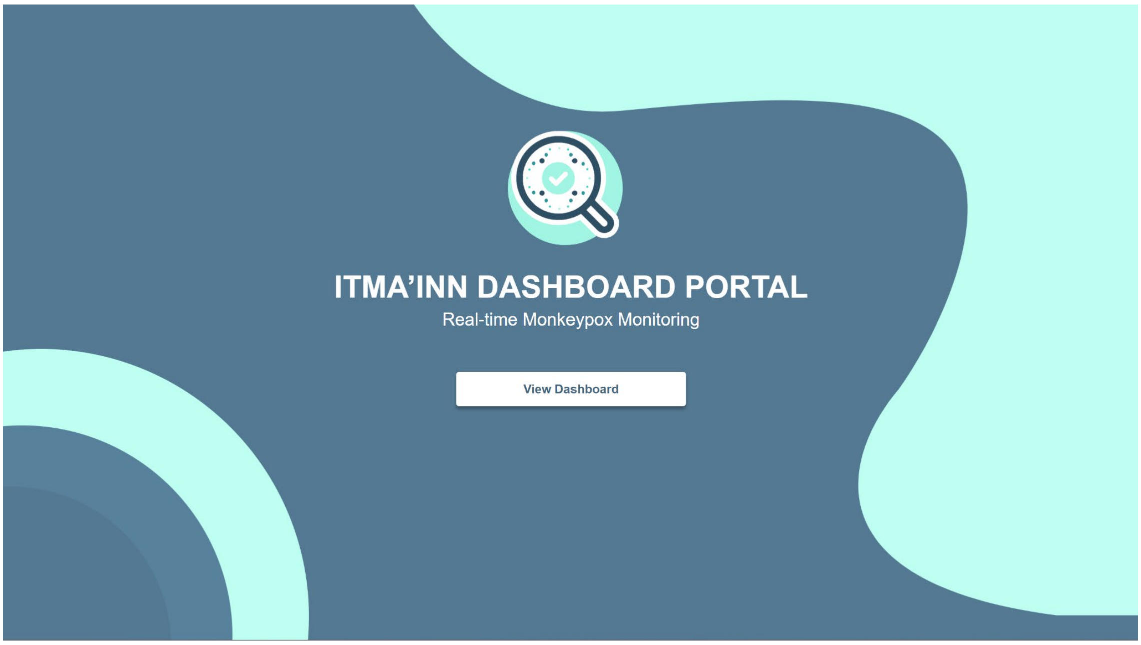

Figure S4. Portal interface of the ITMA'INN system showing the entry point to the real-time monitoring dashboard.

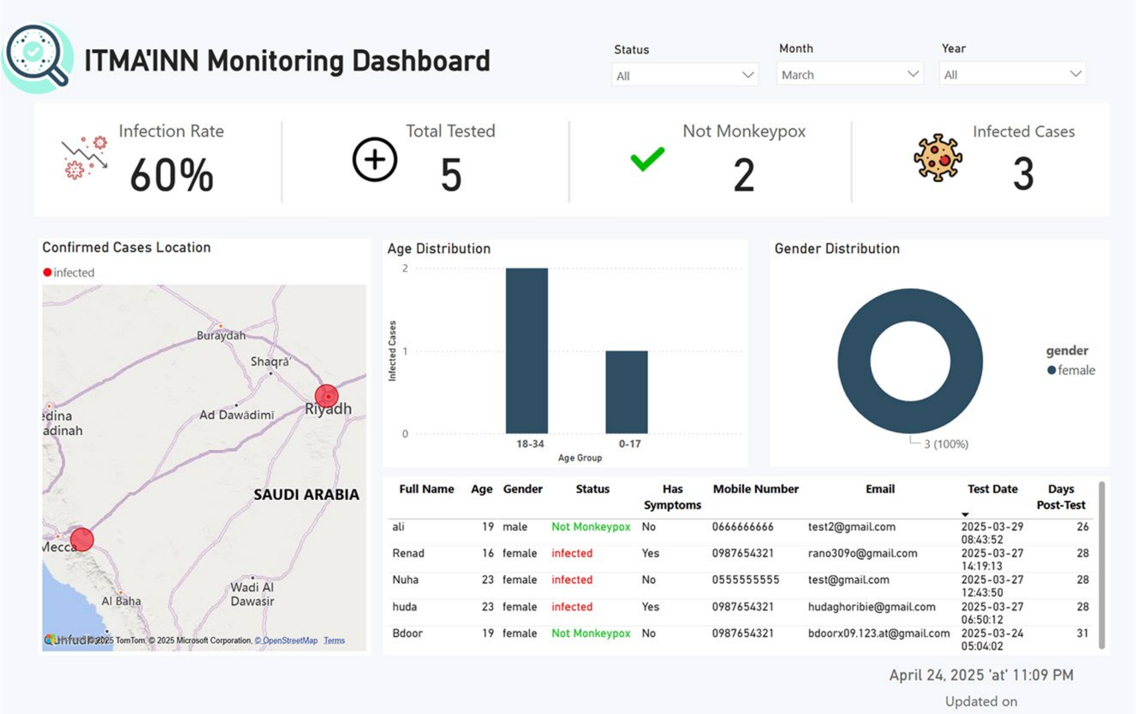

Figure S5. Sample view of the ITMA'INN monitoring dashboard displaying infection rate, demographic distributions, case locations, and patient details in an interactive format.

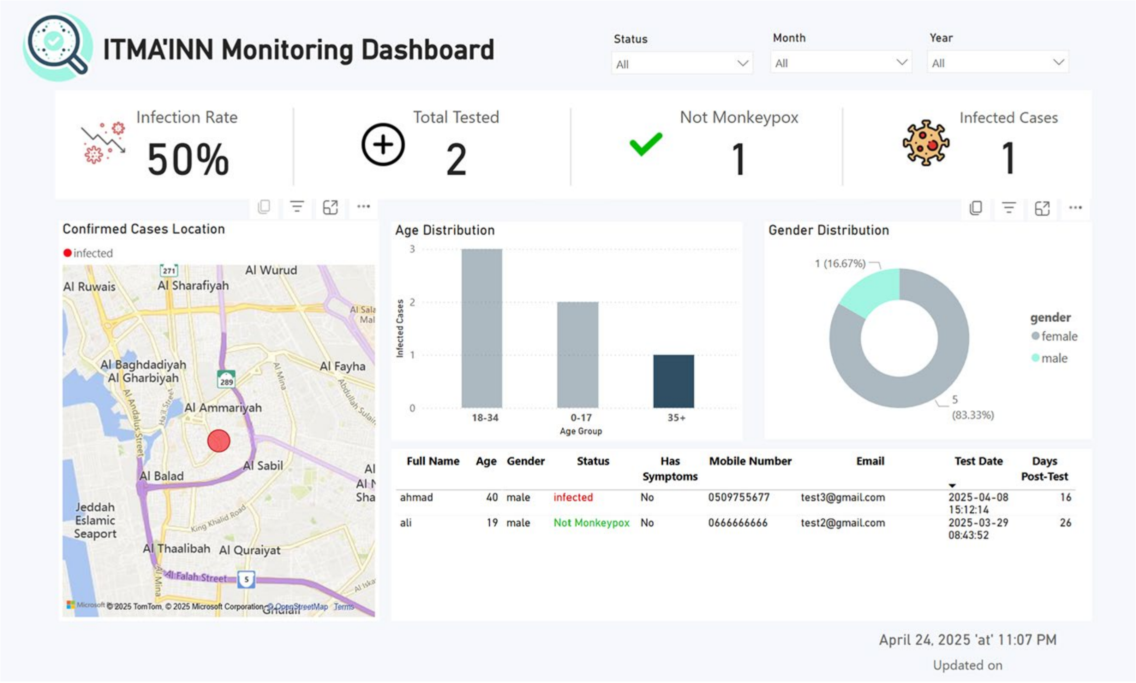

Figure S6. Extended view of the ITMA'INN monitoring dashboard, illustrating data filtered by gender.
